# Mercury speciation in selenium enriched wheat plants hydroponically exposed to mercury pollution

**DOI:** 10.1038/s41598-023-46056-5

**Published:** 2023-11-30

**Authors:** Nithyapriya Manivannan, Maria Angels Subirana, Roberto Boada, Carlo Marini, Mercè Llugany, Manuel Valiente, Laura Simonelli

**Affiliations:** 1 ALBA Synchrotron, Carrer de la llum 2-26, Cerdanyola del Vallès, 08290 Barcelona, Spain; 2 GTS-UAB Research Group, Department of Chemistry, Faculty of Science, Universitat Autònoma de Barcelona, 08193 Bellaterra, Spain; 3 Plant Physiology Group (BABVE), Faculty of Biosciences, Universitat Autònoma de Barcelona, 08193 Bellaterra, Spain

**Keywords:** Environmental sciences, Environmental chemistry, Techniques and instrumentation

## Abstract

Mercury (Hg) pollution in agricultural soils and its potential pathway to the human food chain can pose a serious health concern. Understanding the pathway of Hg in plants and how the speciation may change upon interaction with other elements used for biofortification can be critical to assess the real implications for the final plant-based product. In that respect, selenium (Se) biofortification of crops grown in Se-poor soil regions is becoming a common practice to overcome Se deficient diets. Therefore, it is important to assess the interplay between these two elements since Se may form complexes with Hg reducing its bioavailability and toxicity. In this work, the speciation of Hg in wheat plants grown hydroponically under the presence of Hg (HgCl_2_) and biofortified with Se (selenite, selenate, or a 1:1 mixture of both) has been investigated by X-ray absorption spectroscopy at the Hg L_3_-edge. The main Hg species found in wheat grains was the highly toxic methylmercury. It was found that the Se-biofortification of wheat did not prevent, in general, the Hg translocation to grains. Only the 1:1 mixture treatment seemed to have an effect in reducing the levels of Hg and the presence of methylmercury in grains.

## Introduction

The presence of mercury (Hg) in the environment can be attributed to different activities ranging from coal-fired power plants^1,2^, mining^3,4^, medicine (e.g., dental amalgams^5^, and vaccine production^6,7^). Unfortunately, the release of Hg as a result of these anthropogenic activities causes harmful effects on the environment and to human health^8,9^. Hg species can easily transform and form complexes making Hg speciation in contaminated environment a cyclic process^10–13^. Among all, methylated Hg species cause a major threat due to their high toxicity. The ability of the methylated group to cross the blood–brain-barrier makes it more likely to cause nervous system pathologies in humans^14^. Moreover, in pregnant women, methylmercury species may affect the foetus development and may lead to congenital neural disorders^8,15,16^.

A detailed survey carried out across Europe, sampling mostly agricultural and grazing soils, found that Hg concentration on dry mass ranged from 0.003 to 3.12 mg·kg^−1^^17^. The study indicates that Hg accumulates in those areas of northern Europe where a wet and cold climate favours the build-up of soil organic material. Taking into account that the presence of a Hg concentration above 2 mg·kg^−1^ in the soil is considered an ecological risk^18^, these results highlight the importance of understanding the fate of Hg when it is taken up by the plants and enters the food chain. The Hg species present in soil depend on soil characteristics such as pH and moisture of the soil, the presence of sulphur reducing bacteria, and organic matter content. In general, Hg is present in solid forms as mercurous or mercuric salts which can be reduced into elemental and methylated forms^15^.

The combination of environmental and geological factors together with the plant species define both the level of Hg and its chemical state present in plant-based food^15^. Organic forms and methyl complexes of Hg formed by bacterial methylation are usually absorbed by plants over Hg^0^ at suitable acidic pH^4,11,13^. The bioaccumulation and biomagnification of the methylmercury species in the food chain (crops and fodder)^4,11,13^ produced in Hg polluted soils^19–21^, although less severe than consequences of the Hg pollution in the aquatic environment^19^, is also becoming a health concern^22^.

On the other hand, selenium (Se) is an essential micronutrient which is important for the proper metabolic activity in humans, including reproduction, thyroid hormone metabolism, DNA synthesis, protection from oxidative damage and infection^23,24^. Biofortification of food crops with Se is a viable solution to overcome the Se dietary requirements in regions with Se-poor agricultural lands because plants can convert inorganic Se species to organic ones (e.g., Selenomethionine, Selenocystine, Methyl Selenocysteine) which are more bioavailable for humans^23,25^. This approach could be of particular interest in Europe, where soils present relatively low Se content and suboptimal Se status was reported^26^.

Elements with demonstrated antagonistic effects such as Hg and Se likely may share transporter (e.g., membrane bound metal specific transport proteins), hence the accumulation of one element can potentially reduce the accumulation of the other. The antagonism between Hg and Se^27,28^ has been widely studied in animals^29–31^, and aquatic life^32,33^. Moreover, some birds and cetaceans can demethylate the toxic methylmercury cysteine complex into inert mercury selenide (HgSe) through the formation of an intermediate tetrahedral selenolate complex with selenocysteine (Sec) residues (Hg(Sec)_4_)^34–36^. In plants, the Hg and Se antagonism is gaining attention for various applications including bioremediation and nutritional enrichment. In rice, it has been found that the Se uptake reduces the Hg accumulation by modifying the uptake of other essential nutrients^28,37^. Similarly, in vegetables like radish, it has been reported that Se helps in circumventing Hg uptake^38^. Regarding the metabolism of plants, the sulfur pathway is the most affected pathway under the presence of Se and Hg. This is due to their interaction with transporters and other redox reactions^10,12,28^. Hence, this competing mechanism may have a direct effect on the final Hg concentration accumulated in the plant and on the ratio of the chemical species in crops grown under Hg pollution. In addition, this competition may exert undesirable effects on the outcome of the Se-biofortification process.

In this work, wheat was chosen since it is one of the crops most widely consumed over a wide range of the populations in Europe^39^ and a secondary Se accumulator, i.e. can accumulate Se and show no signs of toxicity over 100 mg Se/kg DW^40^. In addition, wheat is one of the cereal crops which is most largely affected by heavy metal contamination^41–46^. Also, studies have shown that wheat has the ability to accumulate Hg ranging 11–721 mg Hg/kg DW in roots and 2–41 mg Hg/kg DW in leaves when treated with 2.5–25 µM Hg for around a week, even though they show signs of phytotoxicity above 10 µM Hg^47^.

Here we report the Hg speciation study on Se-biofortified wheat grown under Hg exposure. Wheat was cultivated hydroponically to be able to avoid the influence of the soil parameters over the Hg and Se interactions for a clear understanding of the mechanisms involved. The here reported results complement our previous hydroponic studies on Se biofortification wheat^48,49^, and set the basis for future studies in soil culture. Indeed, investigating the effect of Hg in the Se biofortified hydroponic cultivation system will help in better understanding the basic involved mechanisms before increasing the complexity inherent to soil culture media, where the soil characteristics are expected to influence many processes, as the elemental speciation and plant uptake.

The present work aims to study the influence that the different inorganic Se chemical species used for Se-biofortification of wheat crops (sodium selenite and sodium selenate) have over the concentration and speciation of Hg in the different parts of the plant (roots, shoots, grains). Hg L_3_-edge X-ray absorption spectroscopy (XAS) has been used as a direct speciation technique to get information about the chemical state and local coordination structure of Hg. Indeed, XAS provides a powerful tool for in situ chemical speciation, but it is severely limited by poor spectroscopic energy resolution at the Hg L_3_-edge. High energy resolution fluorescence detected XAS (HERFD–XAS) has been here directly compared to standard XAS since it has demonstrated to be a valid quantitative approach for Hg chemical speciation^34–36^.

## Experimental methods

Common wheat (*Triticum aestivum* L. cv. Pinzon) seeds (purchased from Semillas Fito, Barcelona) were germinated in tap water damped filter paper for around 7 days. Afterward, seedlings were transferred to 12 L opaque containers (6 plants per container and 2 containers per treatment to obtain experimental replicates). Half strength Hoagland nutrient solution buffered with MES (2-(N-morpholino) ethanesulfonic acid) to maintain a pH around 6.0 was used (details can be found in Table S1). Se and Hg treatments were applied as described in the Table 1 using mercury(II) chloride (HgCl_2_), sodium selenite (Na_2_SeO_3_), and sodium selenate (Na_2_SeO_4_) as sources for Hg, Se(IV) and Se(VI). The principal Hg and Se chemical species taken up by plants are soluble ionic forms. Furthermore, Hg deposition mainly occurs in the oxidized form (Hg^2+^), and its transformations is associated primarily with the oxidation–reduction potential of the environment and the biological and chemical processes of methylation. Thus, ionic mercury is the first step in the chain and the most accessible to plants. This is the reason why inorganic Hg and Se have been chosen for the treatments. A control group without Hg or Se was also cultivated. For our study, 0.5 µM (0.1 mg kg^−1^) Hg was chosen which is well below the concentration limit imposed for agricultural soils by the current regulations, 1.5 mg kg^−1^^50,51^. A total Se concentration of 25 µM was chosen which is below the reported toxicity threshold value of 100 µM reported for wheat in hydroponic cultures^52,53^. The Se and Hg treatments were applied from the florescence stage of the plant when approximately 1/4 of the inflorescence had emerged and renewed weekly together with the nutrient solution until the plants reached senescence, for 8 weeks. The plants were grown in a controlled growth environment with a relative humidity of ~ 70%, light intensity of 320 μE·m^−2^·s^−1^ and different photoperiods of 12 h of light and 12 h of dark along the plant growth.

**Table 1:** Hg and Se concentrations used for each treatment.

Treatment	Treatment description
Hg(II)	0.5 µM of Hg
Hg + Se(IV)	0.5 µM of Hg + 25 µM Se(IV)
Hg + Se(VI)	0.5 µM of Hg + 25 µM Se(VI)
Hg + Se(Mix)	0.5 µM of Hg + 12.5 µM Se(IV) + 12.5 µM Se(VI)

Matured wheat plants were harvested and divided into roots, shoots, and grains. Samples were oven dried at 65 ºC, ground into a fine powder using an automatic agate mortar and pestle grinder, and stored in airtight tubes until further processing. In previous experiments, the spectra collected on freeze and oven dried samples were compared and no drastic changes were observed between the two methods. Individual plants from different containers were independently characterized in terms of elemental concentration and dry weights to understand how significant the observed differences were considering the natural physiological variability. The Se concentration in different parts of the wheat plants grown under the different Se bio-fortification conditions has been obtained by ICP-MS and is reported in Fig. S1 of the supplementary information.

For shoots and grains, the Hg content from powdered dry samples was determined using a direct mercury analyser, (DMA-80; Milestone Inc., Italy). For the analysis, 2 mg of lyophilized powdered sample were used. The reported error bars correspond to the standard deviation (n = 3) of measurements’ uncertainty. On the other hand, since the Hg level in roots was too high to be measured by the direct mercury analyser, the Hg concentration was determined by X-ray fluorescence (XRF) analysis. In this case, the powders of the roots of 3 replicates were mixed together in equal amount. Detailed information about the procedure followed is reported in the supplementary information (Section "Introduction"). The uncertainty reported corresponds to the standard deviation of the averaged fluorescence counts of the independent channels of the fluorescence detector. The translocation factor is calculated by the ratio between the Hg accumulated in the final part of the plant to the initial part of the plant (e.g., root-to-grain: Hg in grain/Hg in root).

XAS at the Hg L_3_-edge was measured at the BL22 CLÆSS beamline of the ALBA CELLS synchrotron, Spain^54^. Powdered samples coming from the three different replicates of a group were mixed in equal amount and pressed (~ 20 mg) into 5 mm pellets using a hydraulic press. The synchrotron radiation emitted by a wiggler source was monochromatized using a double crystal Si(311) monochromator. The rejection of higher harmonics was done by selecting the appropriate angles and coatings of the collimating and focusing mirrors. Conventional XAS measurements were performed in fluorescence mode at liquid nitrogen temperature using a multi-element silicon drift detector with Xspress3 electronics. High energy resolution Hg L_3_-edge XANES (HERFD) spectra were collected using the CLEAR spectrometer available at the beamline based on Johansson-like dynamical-bent diced-analyser Si crystals for scanning-free energy dispersive acquisition^55^. The Hg Lα_1_ emission was collected using the Si(444) reflection of the analysers working in back-scattering geometry at the liquid nitrogen temperature. The energy resolution estimated from the FWHM of the quasi-elastic line was around 1.4 eV.

Both for standard XAS and HERFD-XANES, no spectral evolution has been identified within the scanning time of 6 min (single repeat) over 1–2 h (full irradiation time). The X-ray absorption spectra were processed using Athena software of the Demeter package^56^ following standard procedures. The linear combination fitting (LCF) analysis was also performed using the Athena software of the Demeter package^56^. The goodness of the fit was obtained by the R-factor ($\sum \left( {data - fit} \right)^{2} /data^{2}$), which is a measure of the mean square sum of the misfit at each data point.

## Results and discussion

**Figure 1:**
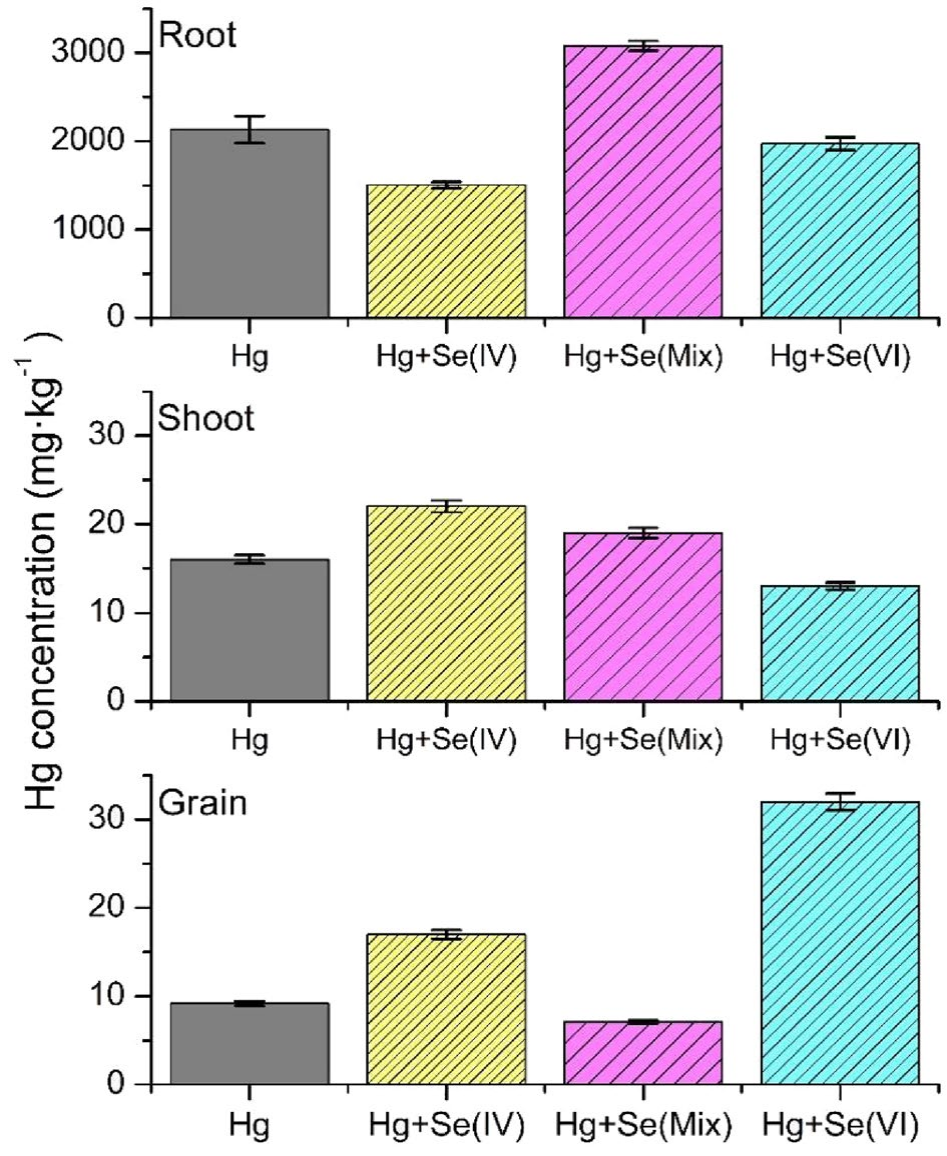
Concentration of Hg (mg·kg^−1^ DW) in different parts of the wheat plant grown under different Se bio-fortification treatments and Hg pollution. Error bar represents standard error from three measurements.

The elemental concentration of Hg in the different parts of the plant is displayed in Fig. 1. The Hg distribution found along the wheat plant resembles the trend reported in previous studies on Hg accumulation in Se-biofortified rice grains^57^. In the present study, all the Hg in the media was available for the plant whereas this is not the case in soils due to the different Hg-soil interactions and plant uptake. This is reflected in the relatively large amount of Hg accumulated along the plant. Regarding the different parts of the plant, roots display the highest Hg accumulation, and among the different treatments, the higher concentration of Hg is found for Hg + Se(Mix), 3080 mg·kg^−1^ dry weight (DW), whereas the other treatments had a slightly lower concentration. For shoots and grains, the Hg concentration drops around two orders of magnitude respect to the amount of Hg found in roots. For grains, the highest Hg concentration is found for Hg + Se(VI), and a significantly lower amount of at least a factor two was found for those treatments including Se(IV), i.e., Hg + Se(IV) and Hg + Se(Mix). The Se concentration found in grains, 132–223 mg kg^−1^, indicates that the Se-biofortification of the wheat grains was achieved as shown in Supplementary Fig. S1.

The Hg translocation factor is reported in Table 2. It was calculated from the ratio of the Hg concentration obtained for different parts of the plants. The results show that Hg is highly accumulated in roots, and very little is translocated to shoots and grains. Plants treated with Hg + Se(IV) show higher translocation from roots to shoots (a factor two respect to other treatments) whereas plants grown with Hg + Se(VI) have higher translocation of Hg from shoots to grains (a factor five respect to other treatments). The translocation from roots to grains for all the treatments was rather small. Interestingly, it is minimized by the mixture of Se species in the feeding, i.e., Hg + Se(Mix).

**Table 2:** Translocation factors of Hg along the plants.

	Hg	Hg + Se(IV)	Hg + Se(VI)	Hg + Se(Mix)
Roots to shoots	0.0075 (± 0.0003)	0.0146 (± 0.0004)	0.0066 (± 0.0008)	0.0062 (± 0.0001)
Shoots to grains	0.58 (± 0.02)	0.77 (± 0.03)	2.46 (± 0.10)	0.37 (± 0.02)
Roots to grains	0.0043 (± 0.0003)	0.0113 (± 0.0004)	0.0162 (± 0.0008)	0.0023 (± 0.0001)

When comparing the different Se treatments, Hg + SeMix shows the highest Hg accumulation in roots and the lowest accumulation in grains. Comparing to the control, Hg + Se(Mix) shows higher accumulation of Hg in roots whereas the other two Se treatments, Hg + Se(IV) or Hg + Se(VI), displays lower Hg accumulation in roots. This suggests that the selenium species used in the feeding influence the accumulation and translocation of Hg and that could be attributed to the different species formation^58,59^. Moreover, this also indicates that the interaction of the different Se species with Hg at the root is not simply additive.

**Figure 2:**
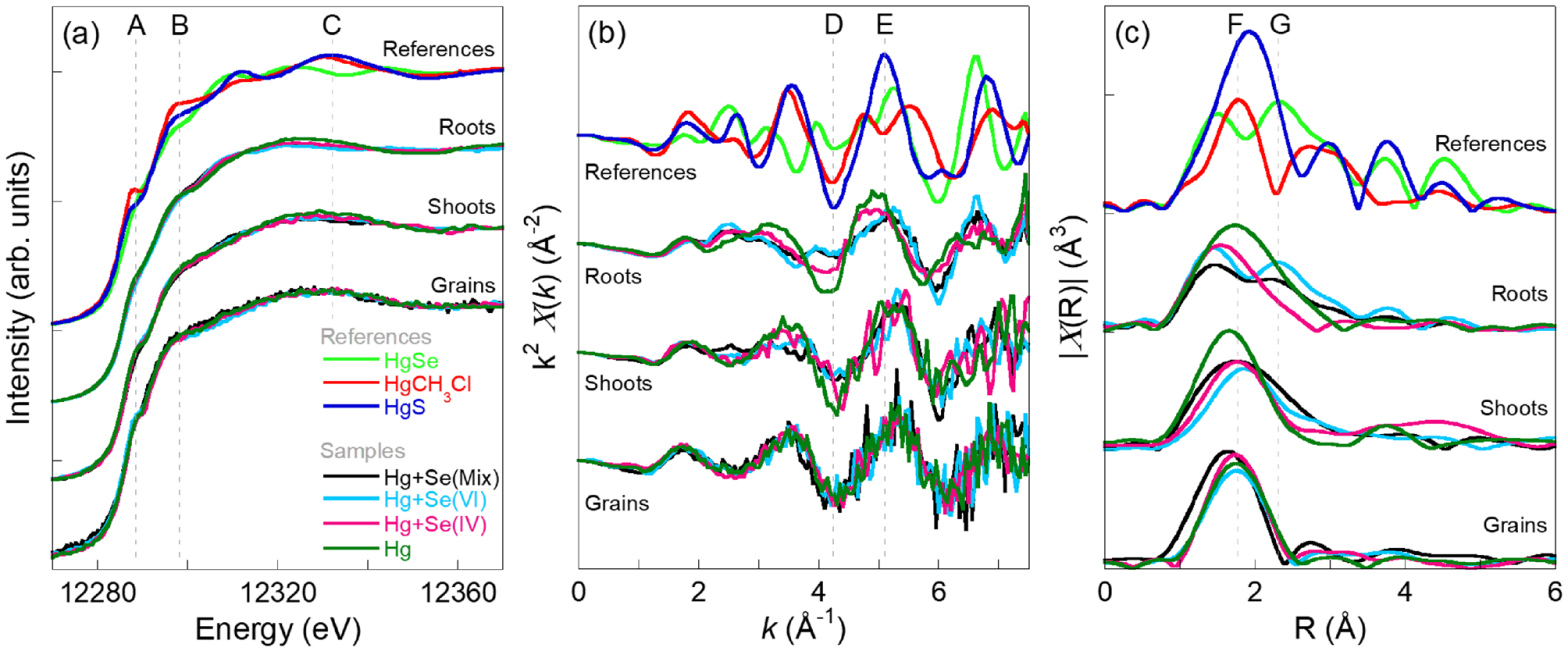
Normalized Hg L_3_-edge XANES (**a**), *k*^2^-weighted EXAFS signal (**b**), and corresponding Fourier transforms (**c**) of spectra acquired over different part of the plants grown with different Se and Hg treatments. The vertical lines are a guide for the eye to identify the main spectral features.

To get a better insight into the translocation of Hg species in wheat plants and the interaction between Hg and Se, Hg L_3_-edge XAS measurements were performed to investigate the chemical state of Hg. Figure 2 shows the comparison of the Hg L_3_-edge XAS spectra collected on roots, shoots, and grains with the most relevant Hg references measured: mercury selenide (HgSe), methylmercury hydrochloride (HgCH_3_Cl), and mercury sulfide (HgS). The spectra of roots, shoots, and grains of wheat plants reveal spectral differences depending on the Se-biofortification treatment and the part of the plant. The spectral profile of each Hg species contained in the sample contributes additively to the total spectrum of the sample, hence, the direct fingerprint comparison and the linear combination fitting analysis using the reference spectra can allow the determination of the species present in the plant samples. The X-ray absorption near edge structure (XANES) region of the spectra is shown in Fig. 2a. The most prominent spectral features in the references set have been labelled A, B, and C. HgCH_3_Cl shows enhanced spectral weight, while HgSe suppressed contributions, at A, B, and C. HgS, instead, presents enhanced contribution at C, while intermediated intensities at A and B. In the XANES region, the relative lower intensity of feature A, B, and C in roots and their progressive increase of intensity going towards the grains suggest a progressive increase of the HgCH_3_ or HgS at the expenses of the HgSe species going from roots to grains.

Although the spectral features of the XANES spectra collected on the samples are not as defined as the ones of the references due to the less ordered coordination environment of Hg in the plants and the probable overlapping contributions, variations in between the samples at the main characteristic features of the reference spectra can be identified. Complementary information can be obtained from the extended X-ray absorption fine structure (EXAFS) region, see Fig. 2b. At around 4.2 (D) and 5.1 Å^−1^ (E) is present the maximum contrast in between the spectra collected on the reference set. By comparing the spectra of the samples with the ones of the references, the EXAFS oscillations characteristic of the HgSe species (suppressed intensity at D) are visible in the roots treated with Hg + Se(IV) and Hg + Se(Mix), while the spectra for Hg control and Hg + Se(VI) suggest the presence of other Hg species such as HgCH_3_ or HgS (enhanced intensity at E). Although less pronounced than in roots since HgCH_3_ or HgS species seem present in higher amount, a similar behaviour is detectable for shoots. The Fourier transforms of the EXAFS signal performed with an Hanning window in between 2 and 7 Å^−1^ are reported in panel c. Despite the small *k* range accessible, two FTs features appear with the Se(IV) and Se(Mix) treatment in the roots, similarly to the FT of the HgSe reference. Going from roots to grains the feature G characteristic of the HgSe reference disappear, while feature F, characteristic of methylated species, become more defined. Indeed, the grains spectra show a FTs similar to the ones of the HgCH_3_Cl reference. In the case of grains, both the EXAFS oscillations and the XANES spectra are less influenced by the Se treatment applied, and the spectral profile at both spectral regions suggest the presence of a higher amount of HgCH_3_ or HgS species irrespective of the treatment. A complementary comparison of the spectra along the plants for each treatment is shown in Fig. S2. This allows to visualize the changes of the Hg speciation along the plant for the different treatments. In the case of Hg control the spectral evolution between the different parts of the plant is minimized clear showing the influence of Se in the Hg speciation along the plant.

**Figure 3:**
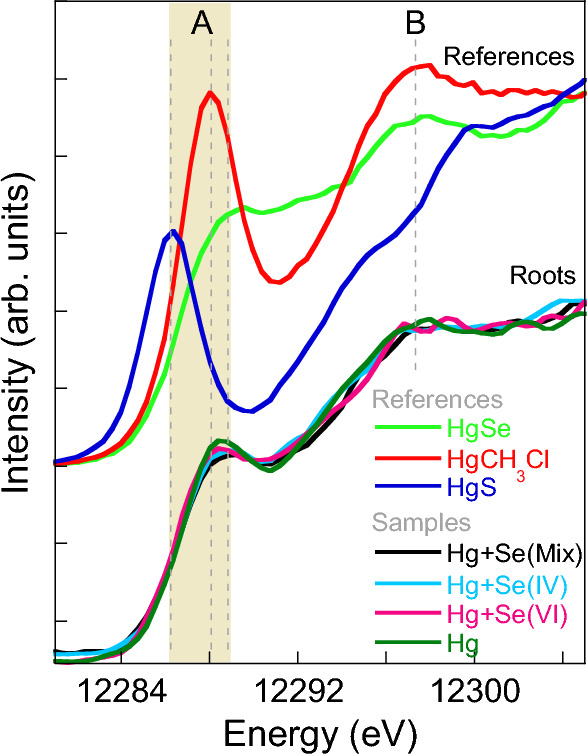
HERFD-XANES spectra collected at the Hg L_3_-edge in the root samples grown with different treatments.

To better resolve the XANES spectral features, high resolution XANES spectra (HERFD) were collected on the root samples, where the concentration of Hg made this approach viable. This technique allows overcoming the intrinsically large core–hole lifetime broadening of the Hg(2p_3/2_) level (5.5 eV)^60^, where a hole is created by exciting across the Hg L_3_-edge by monitoring the 3d_5/2_ final state (2.28 eV)^61^. This approach allows to enhance the spectral differences present as can be clearly seen for the HgS, HgCH_3_Cl, and HgSe references (Fig. 3). In the case of the plant samples, the presence of several spectral contributions broadens the spectra, and partially hides the relative variations. The comparison of the samples and references spectra suggests that the presence of HgS in roots might be minor or negligible since the energy position of the pronounced shoulder at the rising absorption edge A in the samples appears at higher energy, ~ 12,288 eV, than feature A in HgS reference, ~ 12,286 eV (Fig. 3).

**Figure 4:**
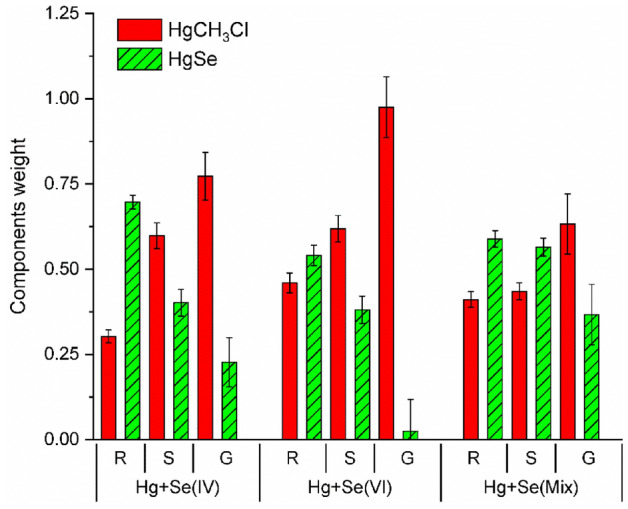
Results from the LCF analysis of the XANES spectra of different parts of the plants (R: roots, S: shoots, G: grain) under different treatments.

To confirm the results from the fingerprint analysis and to better assess the ratio of Hg species present in roots, a linear combination fitting (LCF) analysis of the HERFD-XANES spectra was done. The reference data set is not necessarily the ideal one to describe the data and the reported LCF results aim to a qualitative interpretation describing the main chemical aspects of the Hg species present in the sample data sets and to define trends as a function of the treatment or parts of the plant. Initially all the three reported references have been considered, with the results suggesting a minor or negligible HgS contribution. Then the LCF have been repeated by excluding the HgS contribution, and the corresponding results are reported in Supplementary Table S3 and Fig. S4. They suggest that, in the Se treated sample, the main Hg species present in roots is HgSe. Similarly, LCF of the standard XANES were performed over all the samples considering the three Hg references (HgSe, HgS, and HgCH_3_). The results systematically showed that, in agreement with the HERFD-XANES analysis and the observations reported above, the HgS contribution is absent or negligible in the full set of data. Hence, the HgS reference was excluded in the final LCF analysis shown in Fig. 4 and supplementary Table S4 and Fig. S5. The LCF of the EXAFS signal is reported in the supplementary information Fig. S6, and confirms these trends as shown in Supplementary Fig. S3 and Table S5.

These results should be taken as semi-quantitative since the chemical state of Hg in the plant will be slightly different from the one of the references selected. The coordination environment of Hg-Se in plants will not be as ordered as in the crystalline references used, and the methylmercury group in plants will bind preferentially to thiol groups and amino acids instead to a chlorine atom as in the reference used^62,63^. Despite the limitations of the LCF analysis to quantify the different species, the results shown in Fig. 4 highlight the presence of HgSe in the roots and its progressive reduction in shoots and grains. Complementarily, the highest relative concentration of Hg methylated species is found in grains whereas it is progressively reduced in shoots and roots, likewise results reported in studies about Se biofortified rice^27,28,44,63,64^.

The here reported study was conducted in hydroponic culture. Unlike soil culture, where the presence of different organisms helps in the conversion of Hg species to organic forms^27^, in hydroponics, it is reasonable to assume that the methylated Hg found in the wheat plant has been originated by the transformation or breakdown processes occurring within the plant itself with the help of the different enzymes present in plant cycle. Moreover, bioavailable Hg species, especially methylmercury, can be easily transported from shoots to grains, being the methylated Hg groups insoluble fraction mobile in the nutrient transport of the plants^11,44,57^. Methylmercury is expected to have higher mobility compared with Hg-Se complexes in the plant system^27,28,65^. The formation of a complex between Hg and Se would reduce the fast ligand exchange mechanism of the system due to their stronger bonding. In the same manner, the preference of Hg to form complexes with Se over thiol and sulfhydryl groups would reduce its bioavailability and mobility through the plant metabolism^28^. Anyway, by comparing the different Se + Hg treatments in between them and with the Hg control, it is still necessary to clarify the mechanism that in the Hg + Se(Mix) treatment allow for a partial protection against Hg exposure.

## Conclusions

In summary, the Hg speciation study performed on Se biofortified wheat plants grown hydroponically under Hg exposure allows the determination of the organic and inorganic Hg species accumulated along the plant and as a function of different Se treatments applied. The results show that methylated species are formed both with and without Se-biofortification conditions and that they are the major Hg species stored in the grains. The formation of Hg-Se complexes, present in higher amounts mainly in the roots, is favoured by the Se(IV) treatment, and it seems to affect the Hg translocation to the shoots and grains. The 1:1 mixture of Se(IV) and Se(VI) species in the feeding yields the lowest translocation factor of Hg to the grains. This Se-biofortification treatment inhibits the accumulation of methylmercury in grains offering protection against Hg-induced toxicity to a certain extent. These findings can be applied to understand the consequences of performing Se-biofortification in the presence of Hg contamination. Considering the fact that the Se(Mix) treatment maximize the Se accumulation in grains and minimize the Hg accumulation, it could be suggested that Se(Mix) is the most convenient treatment for Se biofortification when Hg is present in the culture media. The reported results provide a reliable experimental approach which need to be expanded towards field conditions or foliar application for practical purposes.

### Supplementary Information

Supplementary Information.

## Data Availability

The data that support the findings of this study are available from the corresponding authors upon reasonable request.

## References

[1] Morosini C, Terzaghi E, Raspa G, Zanardini E, Anelli S, Armiraglio S, Petranich E, Covelli S, Guardo AD (2021). Mercury vertical and horizontal concentrations in agricultural soils of a historically contaminated site: Role of soil properties, chemical loading, and cultivated plant species in driving its mobility. Environ. Pollut..

[2] Rodríguez-Martin JA, Gutiérrez C, Escuer M, Martín-Dacal M, Ramos-Miras JJ, Roca-Perez L, Boluda R, Nanos N (2021). Trends in soil mercury stock associated with pollution sources on a Mediterranean island (Majorca, Spain). Environ. Pollut..

[3] Esdaile LJ, Chalker JM (2018). The Mercury problem in artisanal and small-scale gold mining. Chem. Eur. J..

[4] Hylander LD, Goodsite ME (2006). Environmental costs of mercury pollution. Sci. Total Environ..

[5] Faheem M, Haji B, Mohammad A (2014). Exposure to mercury from dental amalgams: A threat to society. Mercury Dent. Amalgams.

[6] Baker JP (2008). Mercury, vaccines, and autism: One controversy, three histories. Am. J. Public Health.

[7] De Vries W, Hettelingh J-P, Posch M (2015). Critical Loads and Dynamic Risk Assessments: Nitrogen, Acidity and Metals in Terrestrial and Aquatic Ecosystems.

[8] Nica A, Popescu A, Ibănescu DC (2017). A current problem: Mercury pollution. Curr. Trends Nat. Sci..

[9] Sánchez-Báscones M, Antolín-Rodríguez JM, Martín-Ramos P, González-González A, Bravo-Sánchez CT, Martín-Gil J (2017). Evolution of mercury content in agricultural soils due to the application of organic and mineral fertilizers. J. Soils Sediments.

[10] Alvarez-Fernández A, Díaz-Benito P, Abadía A, López-Millán A-F, Abadía J (2014). Metal species involved in long distance metal transport in plants. Front. Plant Sci..

[11] Arif N, Yadav V, Singh S, Singh S, Ahmad P, Mishra RK, Sharma S, Tripathi DK, Dubey NK, Chauhan DK (2016). Influence of high and low levels of plant-beneficial heavy metal ions on plant growth and development. Front. Environ. Sci..

[12] Singh S, Parihar P, Singh R, Singh VP, Prasad SM (2015). Heavy metal tolerance in plants: Role of transcriptomics, proteomics, metabolomics, and ionomics. Front Plant Sci.

[13] Tangahu BV, Sheikh Abdullah SR, Basri H, Idris M, Anuar N, Mukhlisin M (2011). A review on heavy metals (As, Pb, and Hg) uptake by plants through phytoremediation. Int. J. Chem. Eng..

[14] Sharma BM, Sáňka O, Kalina J, Scheringer M (2019). An overview of worldwide and regional time trends in total mercury levels in human blood and breast milk from 1966 to 2015 and their associations with health effects. Environ. Int..

[15] Humaira, K. T. *Sources and Effect of Mercury on Human Health: A Review* (Project report BRAC University, 2016)

[16] Karita K, Sakamoto M, Yoshida M, Tatsuta N, Nakai K, Iwai-Shimada M, Iwata T, Maeda E, Yaginuma-Sakurai K, Satoh H, Murata K (2016). Recent epidemiological studies on methylmercury, mercury and selenium. Nippon Eiseigaku Zasshi.

[17] Ottesen RT, Birke M, Finne TE, Gosar M, Locutura J, Reimann C, Tarvainen T, Albanese S, Andersson M, Arnoldussen A, Batista MJ, Bel-lan A, Cicchella D, Demetriades A, Dinelli E, De Vivo B, De Vos W, Duris M, Dusza A (2013). Mercury in European agricultural and grazing land soils. Appl. Geochem..

[18] Tóth G, Hermann T, Da Silva MR, Montanarella L (2016). Heavy metals in agricultural soils of the European Union with implications for food safety. Environ. Int..

[19] CookeAndrews J, Atwood DA (2006). Mercury speciation in the environment using X-ray absorption spectroscopy. Recent Developments in Mercury Science.

[20] Navarro A, Biester H, Mendoza JL, Cardellach E (2006). Mercury speciation and mobilization in contaminated soils of the Valle del Azogue Hg mine (SE, Spain). Environ. Geol..

[21] Sysalová J, Kučera J, Drtinová B, Červenka R, Zvěřina O, Komárek J, Kameník J (2017). Mercury species in formerly contaminated soils and released soil gases. Sci. Total Environ..

[22] Ahmed AE, Mosaad AA (2018). Occurrence of trace metals in foodstuffs and their health impact. Trends Food Sci. Technol..

[23] Ellis DR, Salt DE (2003). Plants, selenium and human health. Curr. Opin. Plant Biol..

[24] Zhu YG, Pilon-Smits EAH, Zhao FJ, Williams PN, Meharg AA (2009). Selenium in higher plants: Understanding mechanisms for biofortification and phytoremediation. Trends Plant. Sci..

[25] Wang M, Ali F, Wang M, Dinh QT, Zhou F, Bañuelos GS, Liang D (2020). Understanding boosting selenium accumulation in Wheat (*Triticum aestivum* L.) following foliar selenium application at different stages, forms, and doses. Environ. Sci. Pollut. Res. Int..

[26] Stoffaneller R, Morse NL (2015). A review of dietary selenium intake and selenium status in Europe and the Middle East. Nutrients.

[27] Dang F, Li Z, Zhong H (2019). Methylmercury and selenium interactions: Mechanisms and implications for soil remediation. Crit. Rev. Environ. Sci. Technol..

[28] Li Y, Hu W, Zhao J, Chen Q, Wang W, Li B, Li YF (2019). Selenium decreases methylmercury and increases nutritional elements in rice growing in mercury-contaminated farmland. Ecotoxicol. Environ. Saf..

[29] Cuvin-Aralar ML, Furness RW (1991). Mercury and selenium interaction: A review. Ecotoxicol. Environ. Saf..

[30] Khan MAK, Asaduzzaman AM, Schreckenbach G, Wang F (2009). Synthesis, characterization and structures of methylmercury complexes with selenoamino acids. Dalton Trans.

[31] Khan MAK, Wang F (2009). Mercury-selenium compounds and their toxicological significance: Toward a molecular understanding of the mercury-selenium antagonism. Environ. Toxicol. Chem..

[32] Gailer J, George GN, Pickering IJ, Madden S, Prince RC, Yu EY, Denton MB, Younis HS, Aposhian HV (2000). Structural basis of the antagonism between inorganic mercury and selenium in mammals. Chem. Res. Toxicol..

[33] George GN, MacDonald TC, Korbas M, Singh SP, Myers GJ, Watson GE, O’Donoghue JL, Pickering IJ (2011). The chemical forms of mercury and selenium in whale skeletal muscle. Metallomics.

[34] Manceau A, Gaillot AC, Glatzel P, Cherel Y, Bustamante P (2021). In vivo formation of HgSe nanoparticles and Hg–tetraselenolate complex from methylmercury in seabirds—Implications for the Hg–Se antagonism. Environ. Sci. Technol..

[35] Manceau A, Azemard S, Hédouin L, Vassileva E, Lecchini D, Fauvelot C, Swarzenski PW, Glatzel P, Bustamante P, Metian M (2021). Chemical forms of mercury in Blue Marlin Billfish: Implications for human exposure. Environ. Sci. Technol. Lett..

[36] Minet A, Manceau A, Valada-Mennuni A, Brault-Favrou M, Churlaud C, Fort J, Nguyen T, Spitz J, Bustamante P, Lacoue-Labarthe T (2021). Mercury in the tissues of five cephalopods species: First data on the nervous system. Sci. Total Environ..

[37] Chang C, Chen C, Yin R, Shen Y, Mao K, Yang Z, Feng X, Zhang H (2020). Bioaccumulation of Hg in rice leaf facilitates selenium bioaccumulation in rice (*Oryza sativa* L.) leaf in the Wanshan Mercury Mine. Environ. Sci. Technol..

[38] Shanker K, Mishra S, Srivastava S, Srivastava R, Dass S, Prakash S, Srivastava MM (1996). Study of mercury-selenium (Hg-Se) interactions and their impact on Hg uptake by the radish (*Raphanus sativus*) plant. Food Chem. Toxicol..

[39] Eurostats. *Annual Crop Statistics Handbook* (2020). https://ec.europa.eu/eurostat/cache/metadata/Annexes/apro_cp_esms_an1.pdf

[40] Gupta, M. & Gupta, S. An overview of selenium uptake, metabolism, and toxicity in plants. Front. Plant Sci. Sec. Plant Membr. Traffic Transp. **7**, Article 2074 (2017). 10.3389/fpls.2016.02074 10.3389/fpls.2016.02074 PMC5225104 28123395

[41] Liu WX, Liu JW, Wu MZ, Li Y, Zhao Y, Li SR (2009). Accumulation and translocation of toxic heavy metals in winter wheat (*Triticum aestivum* L.) growing in agricultural soil of Zhengzhou, China. Bull Environ Contam Toxicol.

[42] Li R, Wu H, Ding J, Fu W, Gan L, Li Y (2017). Mercury pollution in vegetables, grains and soils from areas surrounding coal-fired power plants. Sci. Rep..

[43] Li Z, Liang D, Peng Q, Cui Z, Huang J, Lin Z (2017). Interaction between selenium and soil organic matter and its impact on soil selenium bioavailability: A review. Geoderma.

[44] Meng B, Feng X, Qiu G, Liang P, Li P, Chen C, Shang L (2011). The process of methylmercury accumulation in rice (*Oryza sativa* L.). Environ. Sci. Technol..

[45] Wang S, Nan Z, Prete D, Ma J, Liao Q, Zhang Q (2016). Accumulation, transfer, and potential sources of mercury in the soil-wheat system under field conditions over the Loess Plateau, northwest China. Sci. Total Environ..

[46] Wang Y, Dang F, Evans RD, Zhong H, Zhao J, Zhou D (2016). Mechanistic understanding of MeHg-Se antagonism in soil-rice systems: The key role of antagonism in soil. Sci. Rep..

[47] Sahu GK, Upadhyay S, Sahoo BB (2012). Mercury induced phytotoxicity and oxidative stress in wheat (*Triticum aestivum* L.) plants. Physiol. Mol. Biol. Plants.

[48] Xiao T, Boada R, Marini C, Llugany M, Valiente M (2020). Influence of a plant biostimulant on the uptake, distribution and speciation of Se in Se-enriched wheat (*Triticum aestivum* L. cv. Pinzón). Plant Soil.

[49] Xiao T, Boada R, Llugany M, Valiente M (2021). Co-application of Se and a biostimulant at different wheat growth stages: Influence on grain development. Plant Physiol. Biochem..

[50] Commission Regulation (EC) No 1881/2006. *Official Journal of the European Union L 364*, Vol. 49. https://eur-lex.europa.eu/legal-content/EN/ALL/?uri=CELEX%3A32006R1881

[51] Council Directive 86/278/EEC of 12 June 1986. Official Journal of the European Communities, L 181, 4 July 1986. https://eur-lex.europa.eu/legal-content/EN/TXT/?uri=OJ:L:1986:181:TOC

[52] Guerrero B, Llugany M, Palacios O, Valiente M (2014). Dual effects of different selenium species on wheat. Plant Physiol. Biochem..

[53] Li HF, McGrath SP, Zhao FJ (2008). Selenium uptake, translocation and speciation in wheat supplied with selenate or selenite. New Phytol..

[54] Simonelli L, Marini C, Olszewski W, Avila PM, Ramanan N, Guilera G, Cuartero V, Klementiev K (2016). CLÆSS: The hard X-ray absorption beamline of the ALBA CELLS synchrotron. Cogent Phys..

[55] Simonelli L, Marini C, Ribo L, Homs R, Avila J, Heinis D, Preda I, Klementiev K (2023). The CLEAR X-ray emission spectrometer available at the CLAESS beamline of ALBA synchrotron. J. Synchrotron. Radiat..

[56] Ravel B, Newville M (2005). ATHENA, ARTEMIS, HEPHAESTUS: Data analysis for X-ray absorption spectroscopy using IFEFFIT. J. Synchrotron. Radiat..

[57] Li YF, Zhao J, Li Y, Li H, Zhang J, Li B, Gao Y, Chen C, Luo M, Huang R, Li J (2015). The concentration of selenium matters: A field study on mercury accumulation in rice by selenite treatment in qingzhen, Guizhou, China. Plant Soil.

[58] Fortmann, L. C., Gay, D. D., & Wirtz, K. O. Ethylmercury: Formation in plant tissues and relation to methylmercury formation. In *Trace Substances in Environmental Health Conference (USA)*, Vol. 11 117–122 (1978).

[59] Siegel SM, Puerner NJ, Speitel TW (1974). Release of volatile Mercury from vascular plants. Physiol. Plant.

[60] Krause MO, Oliver JH (1979). Natural widths of atomic Kand L levels, K, X-ray lines and several KII Auger lines. J. Phys. Chem. Ref. Data.

[61] Proux O, Lahera E, Del Net W, Kieffer I, Rovezzi M, Testemale D, Irar M, Thomas S, Aguilar-Tapia A, Bazarkina EF, Prat A, Tella M, Auffan M, Rose J, Hazemann JL (2017). High-energy resolution fluorescence detected X-ray absorption spectroscopy: A powerful new structural tool in environmental biogeochemistry sciences. J. Environ. Qual..

[62] Wang F, Lemes M, Khan MAK, Liu G, Cai Y, O’Driscoll N (2011). Metallomics of Mercury: Role of thiol- and selenol-containing biomolecules. Environmental Chemistry and Toxicology of Mercury.

[63] Wang X, Tam NF-Y, Fu S, Ametkhan A, Ouyang Y, Ye Z (2014). Selenium addition alters mercury uptake, bioavailability in the rhizosphere and root anatomy of rice (*Oryza sativa*). Ann. Bot..

[64] Pickering IJ, George GN, Van Fleet-Stalder V, Chasteen TG, Prince RC (1999). X-ray absorption spectroscopy of selenium-containing amino acids. J. Biol. Inorg. Chem..

[65] Zhou J, Liu H, Du B, Shang L, Yang J, Wang Y (2015). Influence of soil mercury concentration and fraction on bioaccumulation process of inorganic mercury and methylmercury in rice (*Oryza sativa* L.). Environ. Sci. Pollut. Res. Int..

