# Supplementary material for: Mercury speciation in selenium enriched wheat plants hydroponically exposed to mercury pollution
Source: Sci Rep. 2023 Nov 30;13:21132. doi: 10.1038/s41598-023-46056-5 (PMC10689832; doi:10.1038/s41598-023-46056-5)
Supplement: Supplementary file 1 — Supplementary Information. [file 41598_2023_46056_MOESM1_ESM.docx]

**Mercury speciation in selenium enriched wheat plants hydroponically exposed to mercury pollution**

Nithyapriya Manivannan^1,2^, Maria Angels Subirana^2^, Roberto Boada^1,2^, Carlo Marini^1^,

Mercè Llugany^3^, Manuel Valiente^2^, Laura Simonelli^1^

^1^ ALBA Synchrotron, Carrer de la llum 2-26, Cerdanyola del Vallès, 08290 Barcelona, Spain

^2^ GTS-UAB Research Group, Department of Chemistry, Faculty of Science, Universitat Autònoma de Barcelona, 08193 Bellaterra, Spain

^3^ Plant Physiology Group (BABVE), Faculty of Biosciences, Universitat Autònoma de Barcelona, 08193 Bellaterra, Spain

Table S1: Half strength Hoagland nutrient solution composition (Arnon and Hoagland 1940)

| **Chemical compound** | **Concentration** |
| --- | --- |
| Potassium nitrate, KNO_3_ | 3.0 mM |
| Calcium nitrate tetrahydrate, Ca(NO_3_)_2_·4H_2_O | 2.0 mM |
| Monopotassium phosphate, KH_2_PO_4_ | 10.0 mM |
| Magnesium sulfate heptahydrate, MgSO_4_·7H_2_O | 0.5 mM |
| Boric acid, H_3_BO_3_ | 3.0 μM |
| Manganese(II) chloride, MnCl_2_ | 2 .0 μM |
| Zinc sulfate hepta hydrate, ZnSO_4_·7H_2_O | 2.0 μM |
| Copper(II) sulfate pentahydrate, CuSO_4_·5H_2_O | 1.0 μM |
| Ammonium heptamolybdate tetrahydrate, (NH_4_)_6_Mo_7_O_24_·4H_2_O | 0.1 μM |
| Ethylenediaminetetraacetic acid, Fe(Na)EDTA | 60.0 μM |
| MES, 2-(N-morpholino)ethanesulfonic acid, C_6_H_13_NO_4_S (Buffer) | 2.0 mM |

**Hg concentration determined by X-ray fluorescence analysis.**

The Hg concentration in roots was determined by X-ray fluorescence (XRF) analysis. The XRF spectra was collected at 12500 eV. The background counts were determined from the XRF spectra collected in the absence of Hg fluorescence (i.e., incident energy set at 12165 eV). For determining the Hg concentration from the integrated signal of the Hg Lα_1_ emission line (9988.8 eV), the Zn Kα emission peak (8638.86 eV) was taken as reference and the appropriate calibration of the XRF signal was done taking into account the Zn concentration determined by ICP-MS (Table S2). The different fluorescence yield of Zn K and Hg L_3_ edges, 0.481 and 0.333, respectively, was taken into consideration.

Table S2: Zinc concentration of samples analysed using ICP-OES (Perkin Elmer Nexton 350D)

| Treatment | Concentration of zinc in mg·kg^-1^ with standard deviation of measurement | | |
| --- | --- | --- | --- |
|  | Root | Shoot | Grain |
| Hg | 307.2 (±15.3) | 130.7 (±7.9) | 189.1 (±8.6) |
| Hg+Se(IV) | 92.6 (±5.3) | 222.3 (±4.3) | 118.4 (±0.8) |
| Hg+Se(VI) | 141.1 (±14.2) | 200.9 (±24.1) | 136.4 (±1.3) |
| Hg+Se(Mix) | 182.3 (±7.9) | 220.9 (±10.2) | 106.6 (±9.4) |

Figure S1 reports the Se content at different parts of the plant. The analysis was carried by ICP-MS (Thermofisher X-series). Samples were previously microwave digested (MARS5 digestor) with nitric acid and hydrogen peroxide at 180 ^o^C with a holding time of 10 minutes. The concentration was determined by external calibration of high purity Se standard along with the addition of internal standards (^45^Sc, ^69^Ga, ^115^In, ^89^Y) for proper monitoring. The concentration of Se is displayed as mean±SD (n=6). Further details can be found elsewhere (Subirana.,2018).


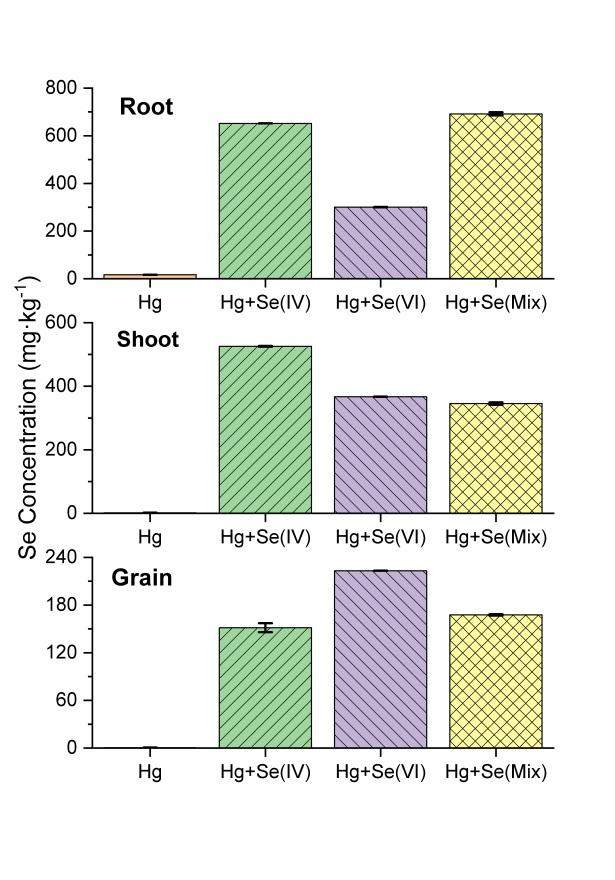


Figure S1: Se concentration (mg·kg^-1^) in different parts of the wheat plant grown under different Se bio-fortification and Hg exposure treatments.


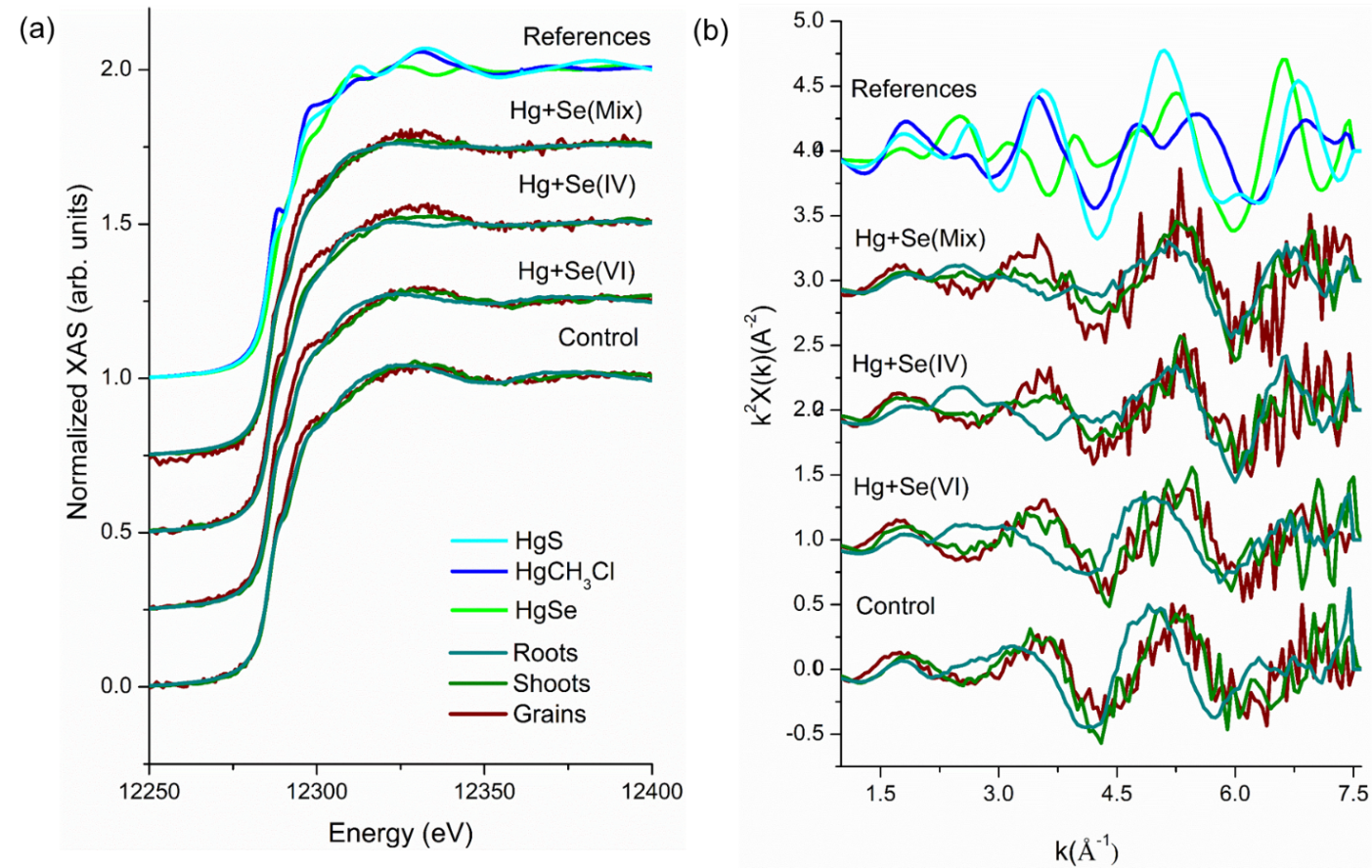


Figure S2: XANES (a) and EXAFS (b) comparison respect to different treatments at various parts of the plant.

Table S3: Results from the LCF analysis of HERFD-XANES measurements in roots. (The values represent the weight of the components present in the system by semi quantitative analysis from LCF. The goodness of the fit was obtained by the R-factor (∑(data-fit)^2^ / ∑data^2^), which is a measure of the mean square sum of the misfit at each data point).

|  | **Hg+Se(IV)** | **Hg+Se(VI)** | **Hg+Se(Mix)** |
| --- | --- | --- | --- |
| **HgCH_3_Cl** | 0.076 (±0.042) | 0.157 (±0.045) | 0.107 (±0.046) |
| **HgSe** | 0.924 (±0.044) | 0.843 (±0.047) | 0.893 (±0.045) |
| ***R-factor*** | 0.010 | 0.012 | 0.012 |

Table S4: Results from the LCF analysis of XANES region of the spectra. R, S and G stand for root, shoot and grain, respectively. (The values represent the weight of the components present in the system by semi quantitative analysis from LCF. The goodness of the fit was obtained by the R-factor (∑(data-fit)^2^ / ∑data^2^), which is a measure of the mean square sum of the misfit at each data point).

|  | **Hg+Se(IV)** | | | **Hg+Se(VI)** | | | **Hg+Se(Mix)** | | |
| --- | --- | --- | --- | --- | --- | --- | --- | --- | --- |
|  | R | S | G | R | S | G | R | S | G |
| **HgCH_3_Cl** | 0.303  (±0.019) | 0.598  (±0.038) | 0.773  (±0.07) | 0.460  (±0.029) | 0.619  (±0.039) | 0.975  (±0.089) | 0.411  (±0.023) | 0.435  (±0.025) | 0.633  (±0.088) |
| **HgSe** | 0.697  (±0.02) | 0.402  (±0.039) | 0.227  (±0.072) | 0.540  (±0.03) | 0.381  (±0.04) | 0.025  (±0.092) | 0.589  (±0.024) | 0.565  (±0.026) | 0.367  (±0.086) |
| **R-factor** | 0.001 | 0.002 | 0.006 | 0.001 | 0.002 | 0.010 | 0.001 | 0.001 | 0.008 |

Table S5: Results from the LCF analysis of the EXAFS region of the spectra. R, S and G stand for root, shoot and grain, respectively. (The values represent the weight of the components present in the system by semi quantitative analysis from LCF. The goodness of the fit was obtained by the R-factor (∑(data-fit)^2^ / ∑data^2^), which is a measure of the mean square sum of the misfit at each data point).

|  | **Hg+Se(VI)** | | | **Hg+Se(VI)** | | | **Hg+Se(Mix)** | | |
| --- | --- | --- | --- | --- | --- | --- | --- | --- | --- |
|  | R | S | G | R | S | G | R | S | G |
| **HgCH_3_Cl** | 0.152  (±0.03) | 0.538  (±0.058) | 0.823  (±0.091) | 0.371  (±0.063) | 0.698  (±0.098) | 0.819  (±0.063) | 0.233  (±0.029) | 0.535  (±0.064) | 0.884  (±0.106) |
| **HgSe** | 0.848  (±0.023) | 0.462  (±0.044) | 0.177  (±0.068) | 0.629  (±0.047) | 0.302  (±0.073) | 0.181  (±0.048) | 0.767  (±0.02) | 0.465  (±0.048) | 0.116  (±0.0079) |
| **R-factor** | 0.090 | 0.403 | 0.574 | 0.528 | 0.668 | 0.349 | 0.118 | 0.350 | 0.509 |


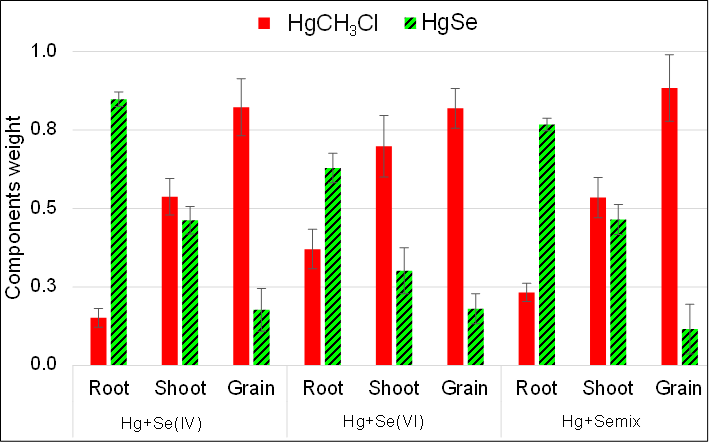


Figure S3: Results from the LCF analysis of the EXAFS region of the spectra.


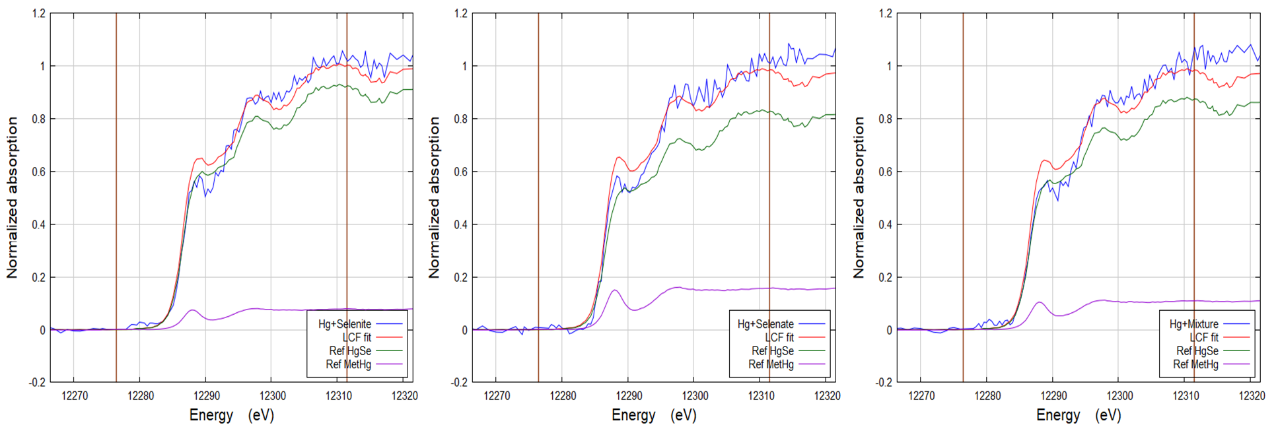


Figure S4: LCF analysis of the HERFD-XANES spectra.


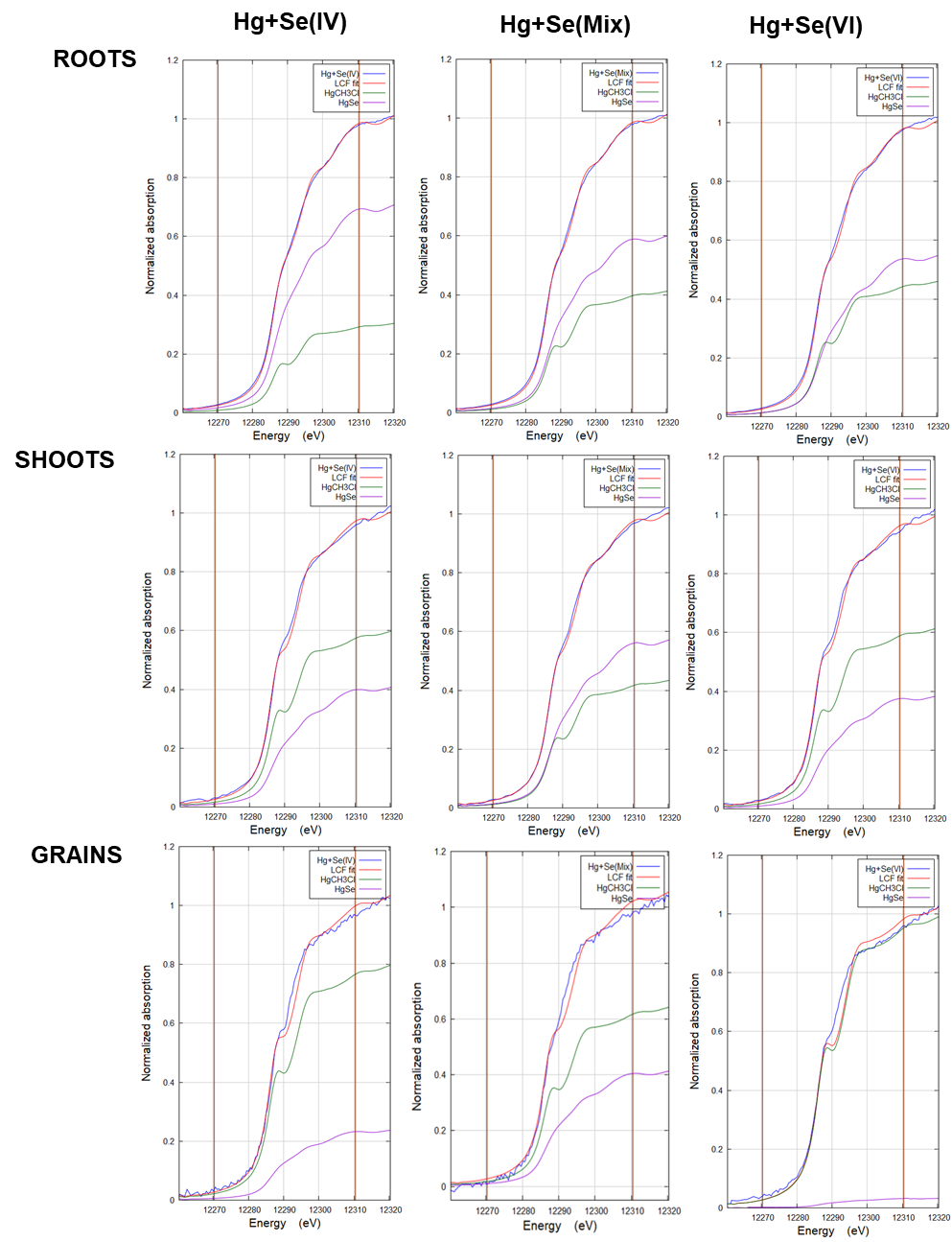


Figure S5: LCF analysis of the XANES region of the spectra.


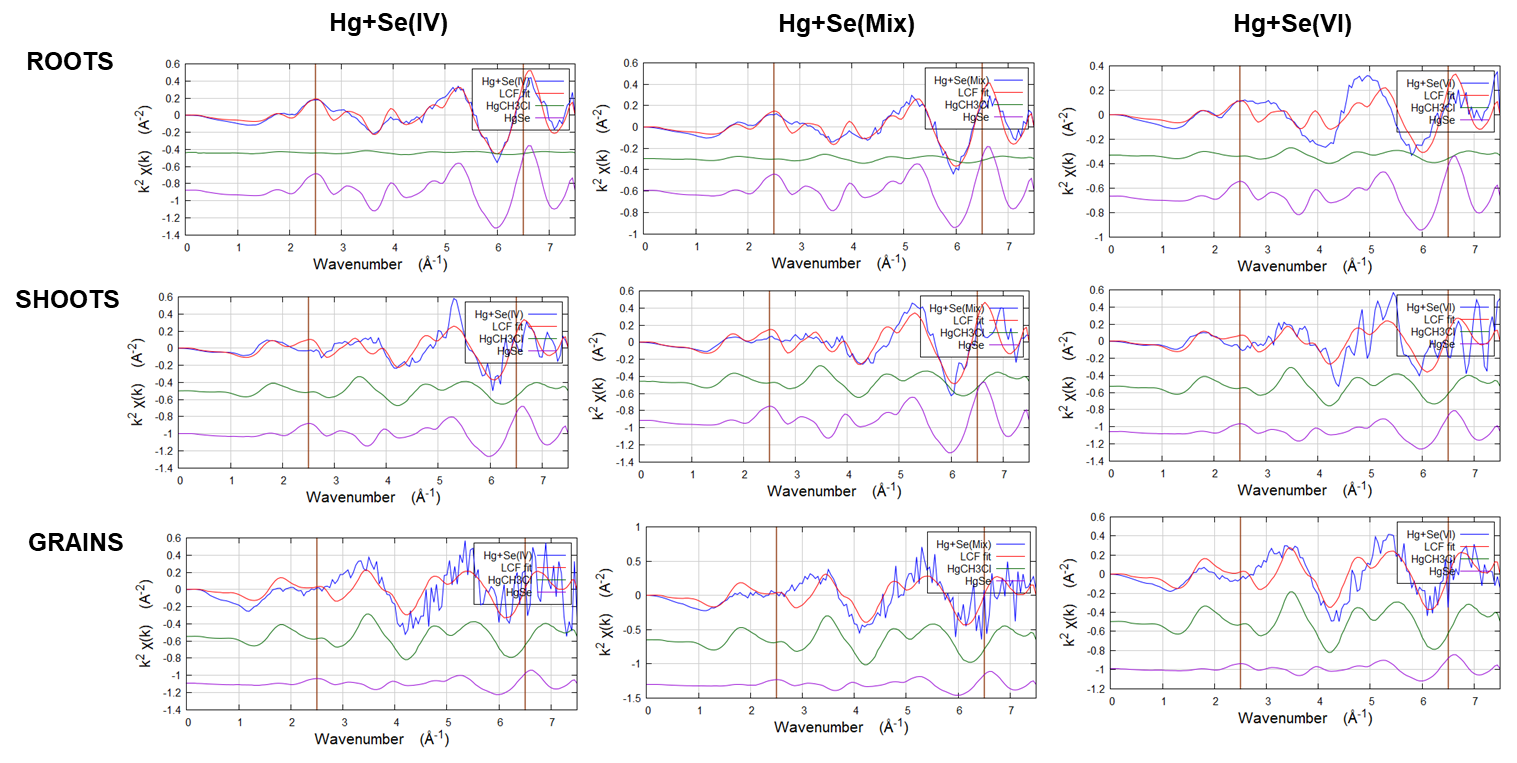


Figure S6: LCF analysis of the EXAFS region of the spectra.

References

Arnon, D.I., and D.R. Hoagland. 1940. “Crop Production in Artificial Culture Solutions and in Soils with Special Reference to Factors Influencing Yields and Absorption of Inorganic Nutrients.” Soil Science 50: 463–85. https://www.cabdirect.org/cabdirect/abstract/19411900537.

Subirana, M. A. (2018). Selenium biofortification of wheat: Distribution and spatially resolved selenium speciation by synchrotron-based techniques. Doctoral thesis, Department of chemistry, Universitat Autònoma de Barcelona.
